# The role of integrating conjugative elements in *Helicobacter pylori*: a review

**DOI:** 10.1186/s12929-018-0489-2

**Published:** 2018-11-29

**Authors:** Langgeng Agung Waskito, Jeng Yih-Wu, Yoshio Yamaoka

**Affiliations:** 1 Department of Environmental and Preventive Medicine, Oita University, Faculty of Medicine, Yufu City, Oita Japan; 2 Institute of Tropical Disease, Universitas Airlangga, Surabaya, Indonesia; 3 Department of Internal Medicine, Kaohsiung Medical University Hospital, Kaohsiung Medical University, Kaohsiung, Taiwan; 4 Department of Medicine, Gastroenterology Section, Baylor College of Medicine, Houston, TX USA; 5 Global Oita Medical Advanced Research Center for Health, Yufu City, Oita Japan

**Keywords:** Plasticity zones, Integrating conjugative elements, Type IV secretion system, *cag* PAI, Virulence factors

## Abstract

The genome of *Helicobacter pylori* contains many putative genes, including a genetic region known as the Integrating Conjugative Elements of *H. pylori* type four secretion system (ICE*Hptfs*). This genetic regions were originally termed as “plasticity zones/regions” due to the great genetic diversity between the original two *H. pylori* whole genome sequences. Upon analysis of additional genome sequences, the regions were reported to be extremely common within the genome of *H. pylori*. Moreover, these regions were also considered conserved rather than genetically plastic and were believed to act as mobile genetic elements transferred via conjugation. Although ICE*Hptfs*(s) are highly conserved, these regions display great allele diversity, especially on ICE*Hptfs*4, with three different subtypes: ICE*Hptfs*4a, 4b, and 4c. ICE*Hptfs* were also reported to contain a novel type 4 secretion system (T4SS) with both epidemiological and in vitro infection model studies highlighting that this novel T4SS functions primarily as a virulence factor. However, there is currently no information regarding the structure, the genes responsible for forming the T4SS, and the interaction between this T4SS and other virulence genes. Unlike the *cag* pathogenicity island (PAI), which contains CagA, a gene found to be essential for *H. pylori* virulence, these novel T4SSs have not yet been reported to contain genes that contribute significant effects to the entire system. This notion prompted the hypothesis that these novel T4SSs may have different mechanisms involving *cag* PAI.

## Background

*Helicobacter pylori* is one of the most successful pathogenic bacteria that colonizes the human stomach, an organ that had previously been considered to be sterile. Colonization of the human stomach resulted in an evolutionary pressure that prompted *H. pylori* to acquire genetic adaptations, leading to high genetic diversity in its genome. The high genetic diversity of bacteria can be attributed to many mechanisms, such as genetic drift, horizontal gene transfer, mutations, and migrations [1]. The rapid evolution process that occurs within the genome of *H. pylori* affects many putative genes that can be divided into three groups: the first group contains genes with variable structures/genotypes depending on the strain. The most well studied example within this group is CagA, which contains a C-terminal repeat segment with a Glu-Pro-Ile-Tyr-Ala (EPIYA) motif and its surrounding region, comprises the EPIYA segment, known as EPIYA-A, -B, and -C/−D, and can discriminate Western-type CagA (with EPIYA-C) and East-Asian-type CagA (with EPIYA-D) [2]. The second group contains the phase-variable genes, the status of which can be altered during the colonization process or in different environments [3]. The best example of genes in this group is the outer membrane protein family. One example is of the blood group antigen binding adhesin (BabA) that is negatively selected during the infection process in the animal models: Rhesus macaques, Mongolian gerbils, and mice [4–6]. Another example is the slipped-strand mispairing mechanism, which can change the functional status of the gene, as is the case in the CT repeat of the outer inflammatory protein (OipA) [7]. The last group contains strain-specific genes, including the *cag* pathogenicity island (PAI), which is among the most well studied and has been reviewed extensively by Backert et al. [8].

In addition to the *cag* PAI, another intriguing putative gene cluster is the Integrating Conjugative Elements (ICEs) of *H. pylori* of type four secretion system (ICE*Hptfs*), which has not been studied as much as other virulence factors such as *cag* PAI and VacA. The fact that this gene cluster has been overlooked can be attributed to inconsistencies and confusion regarding the definition of ICE*Hptfs.* However, with the development of next-generation sequencing (NGS) technology and bioinformatics tools in recent years, we have been able to simplify the definition of ICE*Hptfs*. Besides the more consistent definition of ICE*Hptfs*, current findings highlight the heterogeneity of ICE*Hptfs*, which can be subdivided into ICE*Hptfs*4a/4b/4c and ICE*Hptfs3* [9]. Furthermore, the association of this cluster with the clinical outcomes of infected patients has been reported. In this review, we focus on the current understanding of the ICEs of *H. pylori* in terms of global distribution, heterogeneity, and their association with clinical outcomes.

### Integrating conjugative elements of *H. pylori*

**Fig. 1:**
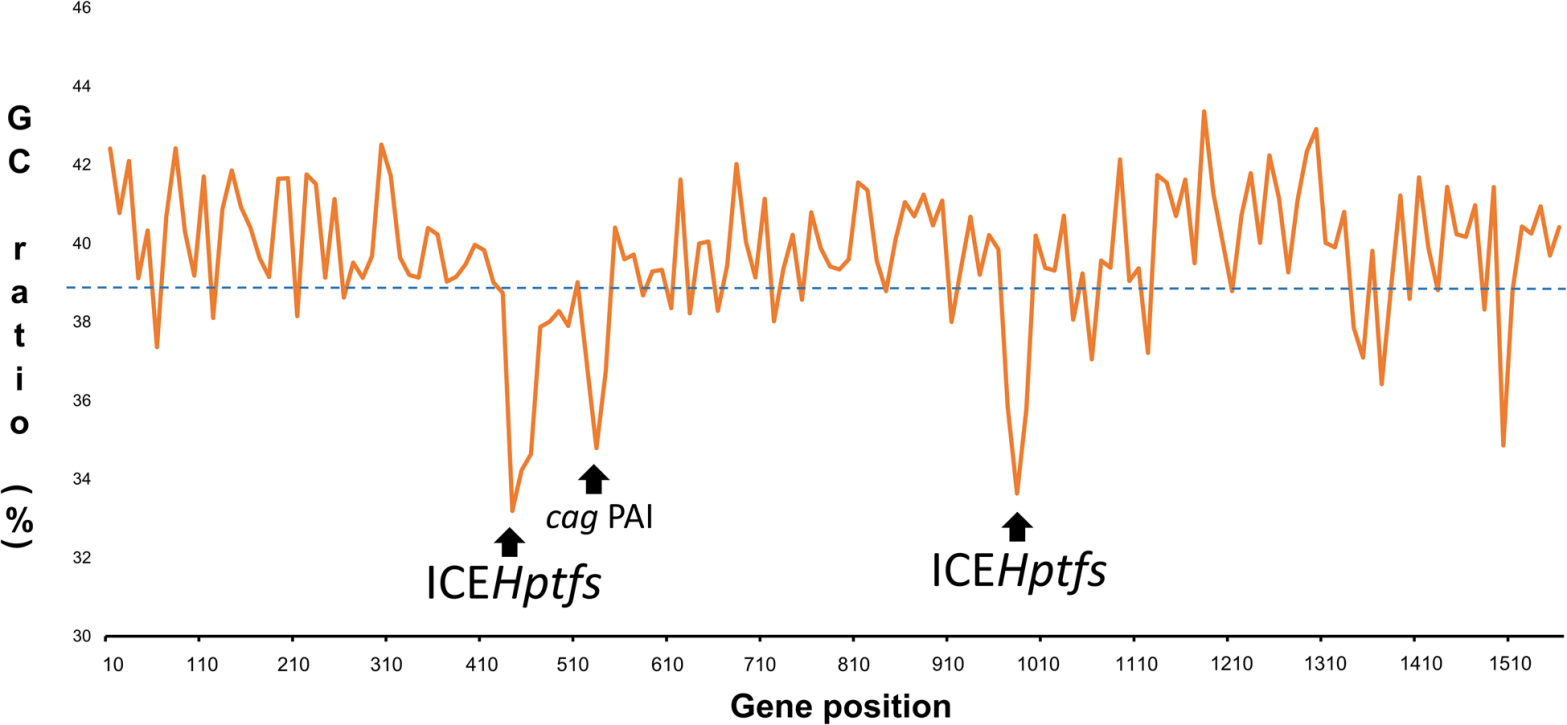
Identification of ICE*Hptfs* based on the G + C content from strain 26,695 (Accession: AE000511.1). The G + C ratio was calculated using EMBOSS [58] of each ORFs. The graph was generated based on the average G + C ratio of 10 ORFs. In the strain 26,695 genome, there are three locations that had significantly low G + C ratio, two of which are ICE*Hptfs* and one is *cag* PAI

Initially ICE*Hptfs* were defined as the plasticity zones or plasticity regions, as they represent potions of the *H. pylori* genome with a considerably lower G + C content (approximately 35%) than the rest of the genome (approximately 39%) (Fig. 1) [10]. The low G + C ratio within these regions were considered a result of horizontal gene transfer from an outside source. Another region within the *H. pylori* genome with low G + C content is *cag* PAI (Fig. 1). However, due to the conserved regions of *cag* PAI between the first 2 fully-sequenced strains, J99 [11] and 26,695 [12], these regions were not considered as plasticity zones. Other low G + C content regions differed between J99 and 26,695; therefore, to address the great diversity between the two strains, these regions became known as plasticity zones [3].

Several years later, the plasticity zones were classified as mobile genetic elements (MGEs) [13]. An MGE is a type of genetic material that can move around within the genome and can be transferred from one species or replicon to another. MGEs can be found in every organism, including bacteria, archaea, and humans [14]. MGEs can have different roles in organism evolution because of the various acquisition sources mediated by HGT. In addition, gene duplication of various components within MGEs has been reported. The MGE can be divided into different types, including transposons, plasmids, and bacteriophages [14]. Transposons are DNA sequences that can move within the genome and include both retrotransposons and DNA transposons. The fundamental difference between retrotransposons and DNA transposons is the requirement of an RNA intermediate in the retrotransposon. Plasmids are a collection of functional genetic modules that are organized into stable, self-replicating entities or “replicons,” which are smaller than the cellular chromosome and usually do not contain any essential functional genes. Bacteriophage refers to a virus that replicates within the chromosome of a bacteria [14].

In the case of plasticity zones within *H. pylori*, the MGEs in the plasticity zones have similar properties to the transposons, and therefore became known as transposon of plasticity zones (TnPZ) (Table 1) [13]. Kersulyte et al. revealed 7 open reading frames (ORFs) of this 16-kb MGE, which are homologues of the VirB system from *Agrobacterium tumefaciens*, including VirB4, VirB7, VirB8, VirB9, VirB10, VirB11, and VirD4, and therefore is considered as a novel type 4 secretion system (T4SS), termed T4SS3 [15]. This cluster was originally identified in the *H. pylori* strain PeCan18B, which was isolated from a patient with gastric cancer in Peru. Interestingly, subsequent studies investigating additional strains from different locations worldwide using polymerase chain reaction (PCR)-Sanger sequence method [13] and NGS [9, 16] showed these MGEs, which were originally thought as “plasticity zones” with high genomic variability [10, 11], were in fact highly conserved. These MGEs were also predicted to have been transferred into the *H. pylori* genome via conjugation as ICEs and are known as ICE*Hptfs* in *H. pylori* [9]. The ICEs on many other bacteria were typically transferred via the following mechanism [17]. First, the element is typically excised from the original chromosome by the recombinase to create circular intermediate. The circular intermediate is then transferred from the donor to the recipient cell via conjugation. Lastly, the ICEs integrate into the recipient cell chromosome via site-specific or non-specific recombination. In the case of ICE*Hptfs4*, the excision step is dependent on XerT recombinase [16], and the conjugation process was dependent on the VirD2 relaxase [18]. The motif for the integration site-specific recombination is most likely AAGAATG (or possibly AAGAAT for ICE*Hptfs*3) [9]. Given the low specificity of ICEs, there are over 100,000 possible integration sites. However, only 40 different integration site motifs have been reported in the case of *H. pylori*. Given that the AAGAATG integration site motif was identified at 550 sites within the *H. pylori* genome, the possible integration sites might be higher [9]. In this regard, the integration site of ICEs in *H. pylori* was considered to possess intermediate specificity.

**Table 1:** The discussed region on major studies about ICE*Hptfs* and its name in those studies

The MGE/T4SS addressed in this review	The MGE/T4SS addressed in [13]	The MGE/T4SS addressed in [16]
ICE*Hptfs3*/T4SS3	TnPZ type 2/TFS3	PZ3/TFS3
ICE*Hptfs4a*/T4SS4a	TnPZ type 1b/TFS3b	PZ1/TFS4
ICE*Hptfs4b*/T4SS4b	TnPZ type 1/TFS3a	n.a.
ICE*Hptfs4c*/T4SS4c	n.a.	n.a.

*n.a.* Not applicable

Based on the newest findings regarding the *H. pylori* MGE, the most recent proposed name of these particular mobile elements is ICE*Hptfs*. In this review, we will refer to these MGE(s), including the TFSS inside of these elements, as ICE*Hptfs* and we will mention the original name from the cited paper. The *cag* PAI indeed had a similar features to ICE*Hptfs,* such as low G + C content, and was flanked by a 31 bp motif representing the integration site [19] and contained the VirB homologue forming *cag* T4SS to translocate CagA [8, 20]. However, upon comparison of the 29 *H. pylori* genomes, the *cag* PAI was classified as a core genome instead of mobile gene clusters, as is the case for ICE*Hptfs3* or ICE*Hptfs4* [21].

### The genetic diversity of ICE*Hptfs* among *H. pylori* strains worldwide

The study of the prevalence of ICE*Hptfs* was initiated in the strains from Costa Rica, isolated from patients with gastritis and gastric cancer in 2000 [22]. Using the dot blot method to determine the presence or absence of ICE*Hptfs* genes in strain J99 (known as the “plasticity region” genes), variation among the Costa Rican strains ranged from 17% (*jhp0940*) to 100% (*jhp0912*) [22]. Subsequent studies used a *H. pylori* genome microarray approach to detect the presence or absence of strain J99 ICE*Hptfs* genes [23, 24]. Those studies investigated a total of 56 [23] and 15 strains [24] worldwide. The prevalence of the J99 ICE*Hptfs* from those 71 strains showed that the prevalence of ORFs in the J99 ICE*Hptfs* varied among the studied strains, except the *jhp0915*, which were present in all studied strains [3]. Although the original purpose of detecting J99 ICE*Hptfs* genes was to screen the candidate genes for genetic markers of clinical outcomes, there were no significant findings until the discovery of a new cluster of T4SS on ICE*Hptfs*. In addition, a subsequent study to investigate the distribution of ICE*Hptfs* using a PCR-based method with 16 representative ICE*Hptfs* ORFs (J99 [11 ORFs], 26,695 [3 ORFs], PeCan18B [1 ORF], and CPY6081 [1 ORF]) to 102 strains variously isolated from Spain, Japan, India, Peru, and Gambia reported ORFs in almost all (92.15%, 94/102) screened strains, with an average of 6 ORFs per strain [13]. The most commonly found ICE*Hptfs* on the *H. pylori* were also reported upon whole genome analysis of 45 [9] and 218 [25] strains of *H. pylori*, for which ICE*Hptfs* were detected in 31/45 (68.8%) and 204/218 (93.5%) strains, respectively. However, our recent study in Indonesia reported the prevalence of ICE*Hptfs* was lower than that in previous reports and ICE*Hptfs* were reported in only 54.3% (56/103) of the analyzed strains [26]. In addition, ICE*Hptfs* were also absent in several CagA types, such as ABBD, AABD, ABCC, and B type CagA. The absence of ICE*Hptfs* in the exclusive CagA genotypes suggests that the distribution of ICE*Hptfs* might be associated with the population genetic of *H. pylori* [26].

**Fig. 2:**
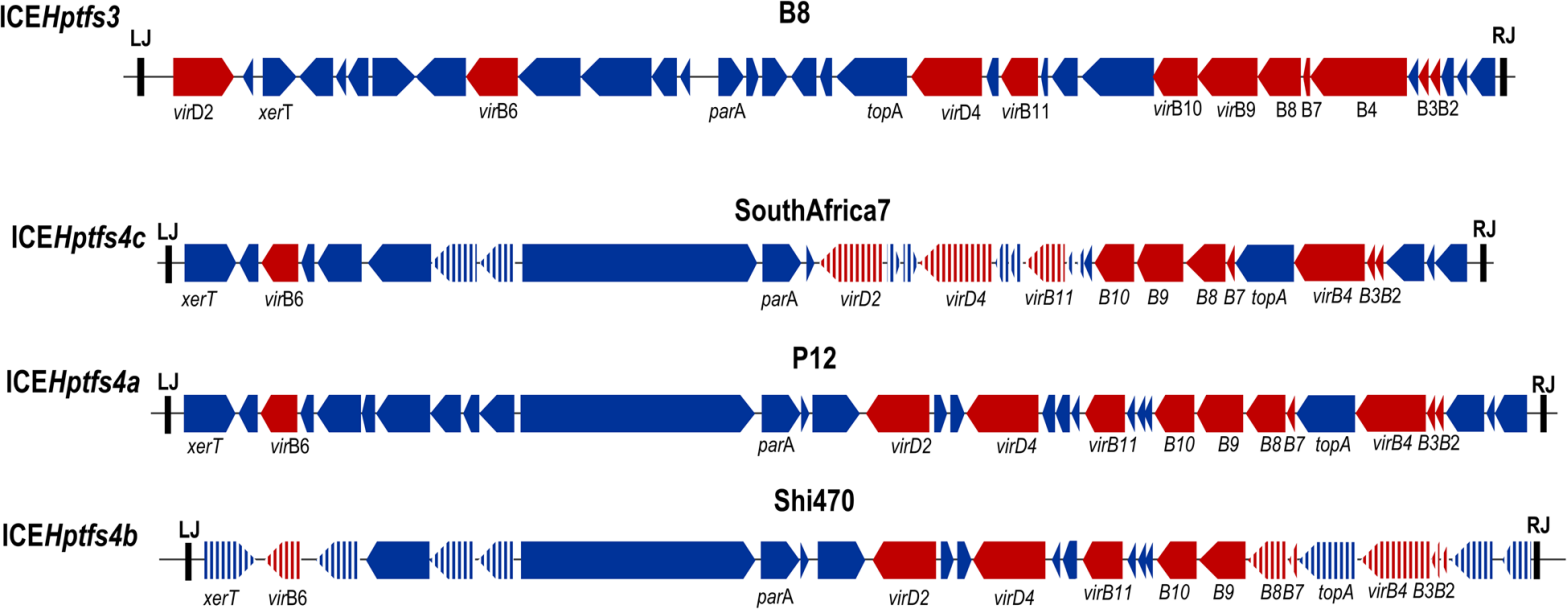
Genetic properties of ICE*Hptfs*3 and ICE*Hptfs*4 based on the Fischer et al. classifications [9]. The red arrow indicates the T4SS-forming genes. The striped pattern indicates a different sequence compared to ICE*Hptfs*4a. ICE*Hptfs*3 and ICE*Hptfs*4 had different overall genetic structures. ICE*Hptfs*4 had three subtypes based of different alleles of the T4SS-forming genes and other accessories genes

Aside from the prevalence of ICE*Hptfs*, which could be locus-dependent, there was an interesting finding on the allele of ICE*Hptfs* within the *H. pylori* genome [9, 13, 16]. The first global analysis of ICE*Hptfs* in 2009 discovered several types of ICE*Hptfs*(s), called as ICE*Hptfs* type 1, type 1band type 2, which also contained a different type of T4SS called T4SS3a, T4SS3b, and T4SS3 (referred to as tfs3, tfs3b, and tfs3 in the study), respectively (Table 1) [13]. In 2010, however, Fischer et al. proposed to change the name of type 1b/1 and type 2 to ICE*Hptfs*4a/4b and ICE*Hptfs*3, respectively (Table 1) [9, 16]. The difference of these genetic elements was determined based on the general structure of ICE*Hptfs* themselves, which showed the location of putative methylase/helicase was directly adjacent to the *virD4* homologue on ICE*Hptfs*3 and the *parA* homologue on ICE*Hptfs*4 [15]. In addition, the type of the T4SS in ICE*Hptfs* could also be distinguished. The analysis of the most conserved region (*virB9*, *virB10*, and *virD4*) of the T4SS in ICE*Hptfs* showed a super lineage between T4SS4 and T4SS3 [9]. In the T4SS4 group, there were three subtypes called T4SS4a, T4SS4b, and T4SS4c (Fig. 2). Both T4SS4a and 4b were most commonly found to contain ICE*Hptfs*4, whereas T4SS4c was only present in the strains from South Africa [9]. As a comparison, the similar genetic cluster occurred because of horizontal gene transfer; the phylogenetic analysis of the *cag* PAI showed there was a linear correlation between the *cag* PAI and the population genetic generated by the multi-locus sequence typing (MLST) [27]. However, the study conducted to analyze 218 *H. pylori* genome sequences showed the there was no correlation in terms of co-occurrence in either the status or type of ICE*Hptfs* with the *cag* PAI [25], suggesting an association to the *H. pylori* population genetic, although the evolution pattern may be different than that of the *cag* PAI.

**Fig. 3:**
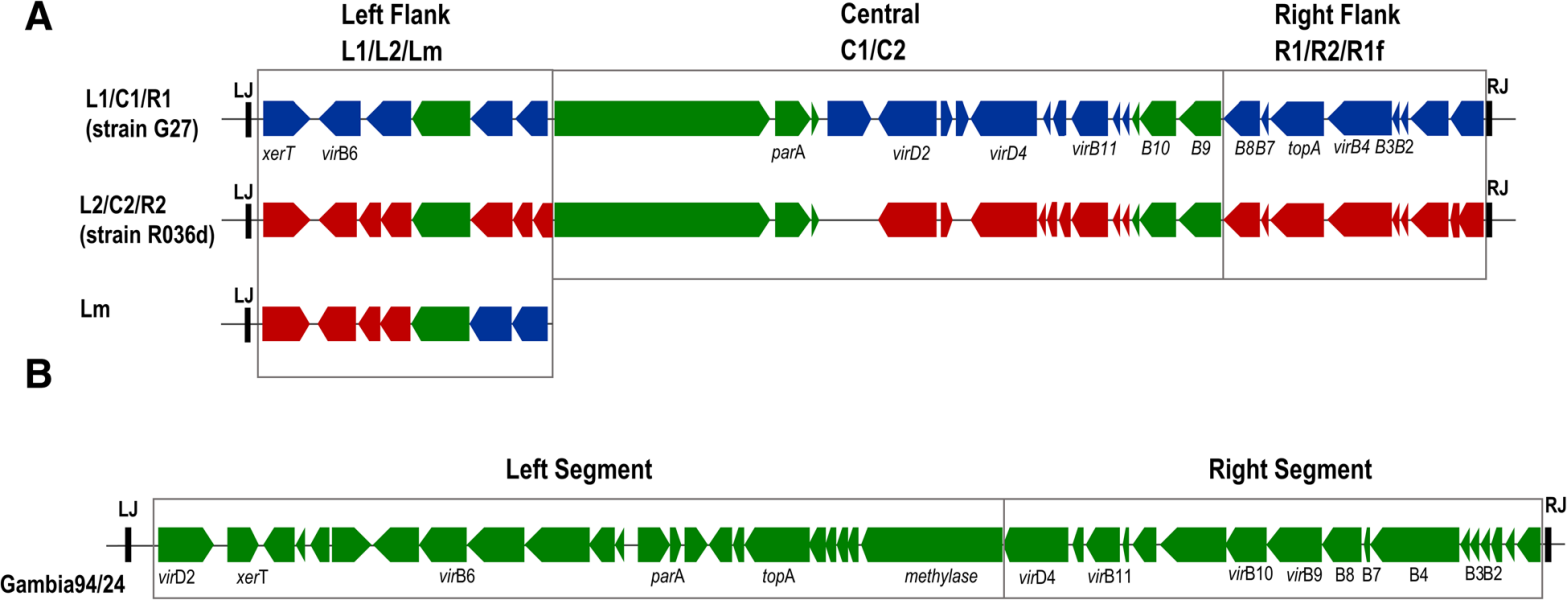
New classification based on orthologue analysis of 218 ICE*Hptfs* genomes adapted from Delahay et al. [25]. **a** The modular subtype of ICE*Hptfs4*. The modular subtype was classified based on three regions: left flank (L1/L2/Lm), central region (C1/C2), and right flank (R1/R2/R1f). The green arrow corresponds to the conserved region, whereas the blue and red arrows correspond to the type 1 (L1-C1-R1/R1f) and type 2 (L2-C2-R2) modules, respectively. The Lm was described as a combination between L1 and L2 on the left flank region. **b** The modular type of ICE*Hptfs3*. Unlike ICE*Hptfs4,* which had a subtype, ICE*Hptfs3* is conserved in almost ICE*Hptfs3*-containing strains with the classification based on the presence/absence of the left segment and the right segment of this region

The analysis of ICE*Hptfs* from hundreds of genome sequences has highlighted another way to classify the various types of ICE*Hptfs*. The classification was created based on the conservative-variable analysis of the structure on a left-central-right segment of the ICE*Hptfs*4 region and on the left-right segment of ICE*Hptfs*3. In ICE*Hptfs*4, the classification was as follows: the left segment as the L1/L2/Lm, central segment as C1/C2, and right segment as the R1/R2/R1f (Fig. 3) [25]. Based on the previous classification, which could differentiate between ICE*Hptfs*4a/4b/4c, utilizing current classification it could be determined as L2-C1-R2 for ICE*Hptfs*4a, L1-C1-R1 for ICE*Hptfs*4b, and Lm-C2-R2 for ICE*Hptfs*4c. Analysis of the type of segments to the population genetic based on the MLST showed a ubiquitous presence of ICE*Hptfs*4, especially the L1-C1-R1 subtype, which may be associated with the ancestral population of *H. pylori* prior to spatial separation [25]. In addition, the C1 subtype was reported to be present in *H. acinonychis,* which is in the same clade as the super lineage of the hpAfrica2 population [28], supporting the existence of an ancient association of this particular element. In contrast, the L2-C2-R2 subtype module had a lower abundance than the type 1 counterpart, suggesting the presence of a different evolutionary history, which may be associated with the recent acquisition and adaptation to a particular isolate of *H. pylori* [25].

In ICE*Hptfs*3, there was no modular subtype of the segments as is the case for ICE*Hptfs*4, which can be differentiated as type 1 and 2. In addition, the distribution of ICE*Hptfs*3 was considerably lower than that of ICE*Htpfs*4, and this particular type was more commonly found as an incomplete cluster than the ICE*Hptfs*4 [25]. This observation was also agreement with our previous findings highlighting that the degree of complete clustering of ICE*Hptfs*3 was lower than that of ICE*Hptfs*4 [26]. In addition, the left segment of ICE*Hptfs*3 was more frequent than the right segment and had a higher co-occurrence with ICE*Hptfs*4 L modules and/or *cag* PAI, suggesting potential differences in stability or temporal acquisition of ICE*Hptfs*3 [25]. Notably, this phenomenon was commonly found in the strain from the hspAmerind population [25]. These data suggest that even ICE*Hptfs*, which were considered to have different evolutionary history than other mobile-like elements like *cag* PAI, still have a characteristic of particular clades of *H. pylori* population, which showed a tendency to play a role as a donor than a recipient [25, 29].

### The role of ICE*Hptfs* in *H. pylori* infection

The MGEs are always transferred to a particular genome with a purpose. One of the reasons behind the transferring process is a fitness advantage, including increased virulence. The most well studied system to confer virulence in *H. pylori* is the T4SS. In fact, the T4SS is found in all known ICEs, genomic islands, and conjugative plasmids of Gram-negative bacteria, except those of *Bacteroides* species [14]. In the case of *H. pylori*, ICE*Hptfs* were predicted to have a genetic advantage as clinical outcome predictors. Indeed, at the initial discovery of this MGE, most of the gene members were hypothetical. However, several genes were predicted to be homologues of the *A. tumefaciens* VirB T4SS components, with other genes being involved in the horizontal gene transfer process, such as XerT, TopA, and ParA [15]. Therefore, most of the studies related to this MGE highlight the association of the genes within these elements to clinical outcome.

The first study was conducted to screen the candidate genes of strain J99 ICE*Hptfs* (“plasticity region” in the study) in the Costa Rican patients, resulting in several genes such as *jhp*0940 and *jhp*0947 as predictors for gastric cancer development and *hp*0986, which was associated with the prevalence of gastritis [22]. The subsequent study involving 200 patients from Brazil with variable clinical backgrounds, including gastric cancer, duodenal ulcer, and gastritis, confirmed that *jhp*0947 was related to the development of gastric cancer with an odds ratio (OR) of 4.14 (95% confidence interval [CI] = 1.47–11.66) [30]. In addition, *jhp*0947 was found to frequently co-exist with *jhp*0949 [31, 32]. Together, these two genes were associated with the induction of interleukin-12 (IL-12) and a higher prevalence of duodenal ulcer [31]. In addition to the induction of IL-12, the *jhp*0947 together with *jhp*0945 and *jhp*0949 induced significantly higher IL-8 and faster apoptosis in the cell lines [33]. The *jhp*0947 gene was found to be homologous to *jhp0938* (*hp0990*) and *jhp0253* (*hp1333*), which all encode hypothetical proteins. However, bioinformatics analysis showed the 5′ region of *jhp*0947 was also homologous to *jhp*0477 (*hp*0528), which is a part of the *cag* PAI (*vir*B9 homologue) and had been identified as an important structural component of the *cag* T4SS. Originally, VirB9 from *A. tumefaciens* contributed to substrate selection and translocation, establishment of channel subunit contacts, and T-pili biogenesis [34]. However, in the case of *H. pylori* homologue in the T4SS4 and/or T4SS3, the function of this gene is yet to be described. Therefore, further studies to elucidate the function are necessary.

In addition, the VirB4 homologue (*jhp0917*/*jhp0918*) of ICE*Hptfs*4b, duodenal ulcer promoting (DupA) was predicted to be important in the development of duodenal ulcer and prevention of gastric carcinoma [35]. *dupA* was screened together with other *virB* homologues within the *H. pylori* genome and was found to be truncated into two genes in the strain J99. The truncated gene was a result of a 1 bp insertion after the position 1385 in strain C145 (GenBank Accession number AB196363), which resulted in a frameshift mutation. However, strain J99 was a rare example in which *dupA* was truncated into two genes. As expected, the screening of *jhp0917* and *jhp0918* revealed that they were always co-existed [35], suggesting that *vir*B4 is a continuous gene of both *jhp0917* and *jhp0918*. A subsequent study in Brazilian strains showed that among 89 strains studied, 86 strains (97%) contained a 1 bp insertion at position 1385 [36], confirming the hypothesis that intact *vir*B4 did not contain a 1 bp insertion downstream of position 1385.

Functional prediction analysis showed that DupA was predicted to be the homologue to *vir*B4 ATPase, which is involved in the DNA uptake/DNA transfer and protein transfer of MGEs. The 5′ terminus of the *dupA*, which is encoded by the *jhp*0917, specifically on the location 3–201, has homology to the FtsK/SpoIIIE family of ATPases [35]. FtsK/SpoIIIE family ATPases are conserved throughout bacteria and are involved in the translocation of DNA and proteins through membrane-spanning pores [37]. In addition, proteins within this family contain a putative ATP-binding P-loop motif, are involved in cell division and peptidoglycan synthesis/modification, and have been implicated in intercellular chromosomal DNA transfer. The central region of *dupA*, which is encoded by *jhp*0917 5′–*jhp*0918, specifically at position 203–610, showed homology to the TraG/TraD family protein [35]. TraG-like proteins are potential NTP hydrolases (NTPases) that are essential for DNA transfer in bacterial conjugation and may mediate the interaction between DNA processing and mating formation systems [38].

#### The importance of cluster genes rather than a single gene

The association between the pathogen and the infected host involves a complex interaction between the bacterial genetics, host vulnerability, and the environment of the infection. With respect to bacterial genetics, most of the reported findings highlight that a single gene or cluster of genes is responsible for the development of severe clinical outcomes of the infected individual. In *H. pylori*, the most well studied virulence gene is *cagA*, which encodes the oncogenic protein CagA. CagA is a member of *cag* PAI, a T4SS complex that can form a pilus to surround *H. pylori*, allowing for the transfer of the CagA protein into the host cell [8]. There are many studies showing the association of *cagA* to the prevalence of gastric carcinoma in the Western population [39–41]. However, nearly all of the isolated strains from East-Asian countries contained *cagA,* and therefore the presence or absence of CagA alone does not discriminate the clinical outcomes of infected individuals [2]. Hence, it was proposed that the heterogeneity of the C-terminus of CagA, which includes repeat segment containing EPIYA motif and its surrounding region, comprises the EPIYA segment, known as EPIYA-A,-B, and -C/−D, and can discriminate between Western-type CagA and East-Asian-type CagA, respectively [2]. East-Asian-type CagA has a higher affinity for the SH2 domain, which can result in a worse cascade output directly following the initial infection [42]. However, our recent study exploring the correlation of CagA heterogeneity status with the clinical outcome did not show a promising result [43–45]. Our observation in Indonesia showed that individuals infected with East-Asian-type CagA strains had even lower inflammation scores than those infected with Western-type CagA strains [44]. In addition, reports from two locations in Thailand with different gastric cancer incidences further contradicted previous findings, as individuals infected with Western-type CagA strains were found to have significantly higher antral activity than those with the East-Asian-type CagA strains [45]. Moreover, our observation in Mongolia, the second highest gastric carcinoma prevalence based on age standardization rate (ASR); GLOBOCAN, 2012 (http://globocan.iarc.fr) (ASR = 47.4/100,000 men), showed our developed East-Asian specific CagA antibody [46] had negative results in most of immunohistochemistry biopsy specimens [43], suggesting the most CagA type in Mongolia was Western type. These data suggest that the CagA is still essential, but in certain areas, a complex interaction between infected individuals and the pathogen to generate a clinical outcome may not be attributed to a single gene. Therefore, it is interesting to investigate additional genes in close proximity that may be involved (e.g. *cag*A and *cag* PAI, *dupA* cluster) or genes from another system that may interact in an epistatic manner.

Initial observations of *cag* PAI intactness to allele diversity of *vacA* showed the intact contiguous *cag* PAI was frequently found in patients with peptic ulcer diseases (78%) and gastric carcinoma (73%) and that this frequency was significantly higher than that in the gastritis patients (40%, *p* < 0.01) [47]. Among the virulence factors, there was an association between intact *cag* PAI and both the *vacA* s1 allele and m1 allele (*p* < 0.005 and *p* = 0.05, respectively) [47]. A subsequent study in Sweden showed that *H. pylori* isolates containing all the genes within the *cag* PAI induced high IL-8 production in AGS cells, a gastric cancer cell line. Additionally, the presence of intact *cag* PAI was reported to have more than five-times higher risk of developing more severe gastroduodenal diseases than the absence of *cag* PAI (95% CI = 1.5–17.4) [48]. Another observation of the association between intact *cag* PAI to the clinical outcome was also reported in Iran [49]. The patients infected with intact *cag* PAI strains were reported to have more severe atrophy than those with non-intact *cag* PAI strains [49]. Our observation in Vietnam similarly showed that there was an association of intact *cag* PAI *H. pylori* with patients with peptic ulcers, as all of the enrolled peptic ulcer patients were infected with intact *cag* PAI *H. pylori* [50]. In addition, patients infected with intact *cag* PAI had higher inflammation scores in the antrum, corpus, and upper lesser curvature [50]. In our recent study, we observed that in Indonesia, the *cag* PAI was identified in almost all Indonesian *H. pylori* isolates (99%), but was associated with various clinical outcomes. However, after detailed analysis of the gene functionality, we found that several genes contained either a premature stop codon or a frameshift mutation within the *cag* PAI genes. Therefore, we consider a region containing a non-functional gene to represent non-intact *cag* PAI. These findings suggest that the intactness of *cag* PAI does not solely depend on the presence or absence of the *cag* PAI-forming genes and also depends on the functional status of the forming genes [26]. These findings suggest that the importance of virulence factors may be determined by a cluster of related genes, rather than a single gene in isolation.

The concept of a cluster of the genes, which may more accurately predict the clinical outcome of the infected individual, is also relevant to ICE*Hptfs* genes. In 2005, our group discovered *dupA*, which had a strong positive correlation with the prevalence of duodenal ulcer, but a negative correlation with gastritis atrophy, intestinal metaplasia, and gastric cancer [35]. Indeed, the subsequent systematic review and meta-analysis involving 17 studies with 2466 patients of *dupA* showed a positive correlation between *dupA* and the prevalence of duodenal ulcer in the general population (OR = 1.41, 95% CI = 1.12–1.76), while subsequent analysis in the Asian population showed an OR = 1.57 (95% CI = 1.19–2.06) [51]. However, in the Western population, *dupA* did not show any correlation with the prevalence of duodenal ulcer (OR = 1.09, 95% CI = 0.73–1.62) [51].

Notably, there were several inconsistent results in studies that followed the discovery of *dupA*. *dupA*, a homologue of VirB4 from ICE*Hptfs*4b [9], was reported to have an extra 600 bp on the 5′ end of the original *dupA* gene (*jhp*0917-*jhp*0918). The variant was referred to as *dupA* long type [52], with two different functional status characterized as long intact and long non-intact depending on the presence/absence of a 1 bp insertion at position 1385 in strain C145, resulting in a frameshift mutation [35]. In the Okinawan population in Japan, long-intact *dupA* significantly increased the risk of infected individuals to develop gastric cancer and ulcers rather than gastritis by more than 3-fold and 4-fold (OR = 3.3, 95% CI = 1.55–7.24 and OR = 4.14, 95% CI = 1.23–13.94), respectively [52]. In addition to the observation of a 1 bp insertion, the *dupA* was reported to have another allele polymorphism with an adenine deletion at position 1131 and an adenine insertion at position 1426. These insertions and deletions can lead to premature stop codons, which may produce a non-functional gene. We observed that this allele polymorphism leading to intact *dupA* (an allele without any insertions and deletions leading to a premature stop codon) was associated with the increase in mucosal inflammation, but an overall decrease in mucosal atrophy. Moreover, the intact *dupA* was negatively associated with gastric carcinoma [53]. These findings suggest that the long-intact *dupA* was more reliable as a clinical outcome predictor than the short type *dupA*.

Notably, *dupA* is surrounded by several genes such as *virB8*, *virB9*, *virB10*, and *virB11* which can form the T4SS, named as T4SS4b, which lies within ICE*Hptfs4b*. Therefore, once all the components necessary for forming the T4SS have been identified, we will gain a better understanding of using this biomarker to predict the clinical outcome of infected patients. Our epidemiological observation of the *dupA* and its cluster forming the T4SS showed that the complete *dupA* cluster was significantly correlated with the prevalence of duodenal ulcer in the US population [54]. In addition, individuals infected with *H. pylori* containing the intact *dupA* cluster showed a higher atrophy score in both the antrum and corpus. In the in vitro model, isolates with intact *dupA* cluster *H. pylori* could induce significantly higher IL-8 production in both gastric epithelial cells and the gastric cancer cell line MKN45 [54]. Our latest observation in Indonesia showed the intact ICE*Hptfs4b* was the most associated with increased inflammation in the antrum compared with ICE*Hptfs*-negative status [26]. In addition, in combination with the *cag* PAI, patients infected with *H. pylori* containing both intact *cag* PAI and ICE*Hptfs4b* had the highest inflammation, both in the antrum and corpus [26]. Furthermore, in *H. pylori* isolates from pediatric patients, the *virB4*-like gene *dupA* was not associated with any differences in IL-8 production and NF-κB phosphorylation in infected gastric cells. However, the complete cluster of *dupA H. pylori* isolates, which encoded T4SS4b, induced significantly higher IL-8 production in infected gastric epithelial cell lines [55]. These findings when taken together suggest several points. Firstly, *dupA* alone is an essential factor for determining the clinical outcome of an infected individual. This has also been shown using in vitro studies [35], although there are several discrepancies between different populations. Second, *dupA* and its other homologues from ICE*Hptfs4*a/*3* play a role in the formation of the T4SS, and are therefore more predictive of disease than a single gene.

In addition to the role of *dupA*, which belongs to ICE*Hptfs4b*, a recent study had reported a role of ICE*Hptfs3* in the *H. pylori* infection process. One of the ICE*Hptfs* forming genes was predicted to encode cell translocating kinase A (CtkA). CtkA is a protein that induces a pro-inflammatory response within infected host cells [56]. Despite the previous understanding that CtkA was considerably more variable in different geographic populations, a recent study showed that CtkA was encoded by one of the genes within ICE*Hptfs3*, which are located closer to *xer* and *virD2* toward the end of the ICE in *H. pylori* and *H. cetorum* [57]. In addition, CtkA-induced expression of pro-inflammatory cytokines was dependent on ICE*Hptfs3*, but independent of the *cag* PAI. The induction of pro-inflammatory cytokines within the infected cell lines was mediated by the activation of NF-κB [57]. These data confirm the importance of ICE*Hptfs3* during *H. pylori* infection, in particular through CtkA as one of the substrates of this genetic island.

## Conclusion

Since the discovery of this particular gene region in the *H. pylori* genome, there have been a variety of proposed names including the plasticity region, plasticity zones, *Tn*PZ, and ICE*Hptfs*, which has led to the confusion regarding the terminology. Based on new findings regarding this gene cluster, the most relevant name is ICE*Hptfs.* With the increasing number of available genome sequences, it has been reported that this region is commonly present in *H. pylori* genome with some variability in the different geographic areas. In addition, ICE*Hptfs* were also reported to have novel T4SS(s) with functions related to virulence. However, the function of each component forming the full set as well as the structure of this novel T4SS are yet to be described. Therefore, future studies to elucidate the genetic components of the T4SS, the structure of the formed T4SS, and the role of each gene in T4SS function are necessary. Unlike the *cag* PAI, which has the CagA gene that has been shown to be essential for virulence, no genes with significant effects on the entire system have been identified within these novel T4SSs. This condition leads to the hypothesis that these novel T4SSs may have different mechanism involving the *cag* PAI.

## Abbreviations

ASR: Age standardization rate
CI: Confidence interval
CtkA: Cell translocating kinase A
ICE: Integrating conjugative element
IL: Interleukin
MGE: Mobile genetic element
MLST: Multi locus sequence typing
NGS: Next generation sequencing
OR: Odd ratio
ORF: Open reading frame
PAI: Pathogenicity island
PCR: Polymerase chain reaction
T4SS: Type IV secretion system
TnPZ: Transposon of plasticity zones
